# *N*′-(3-Bromo-5-chloro-2-hy­droxy­benzyl­idene)-2*H*-1,3-benzodioxole-5-carbo­hydrazide

**DOI:** 10.1107/S160053681201433X

**Published:** 2012-04-06

**Authors:** Jiao Wei, Hong-Yan Ban, Xiao-Zhi Sun

**Affiliations:** a School of Chemical Engineering, University of Science and Technology Liaoning, Anshan 114051, People’s Republic of China

## Abstract

The asymmetric unit of the title hydrazone compound, C_15_H_10_BrClN_2_O_4_, contains two independent mol­ecules. The dihedral angles between the benzene rings are 38.7 (3)° in one mol­ecule and 24.3 (3)° in the other. Both mol­ecules exist in *trans* conformations with respect to the C=N double bonds of the central methyl­idene units. Intra­molecular O—H⋯N contacts are observed in both mol­ecules, forming *S*(6) rings. In the crystal, mol­ecules are linked through N—H⋯O hydrogen bonds into chains along the *a* axis.

## Related literature

For the biological activity of hydrazones, see: Zhong *et al.* (2007 ▶); Raj *et al.* (2007 ▶); Jimenez-Pulido *et al.* (2008 ▶). For related structures, see: Ban (2010 ▶); Ban & Li (2008*a* ▶,*b* ▶); Li & Ban (2009*a* ▶,*b* ▶); Yehye *et al.* (2008 ▶); Fun *et al.* (2008*a* ▶,*b* ▶); Yang *et al.* (2008 ▶); Ejsmont *et al.* (2008 ▶); Yang (2006 ▶). For hydrogen-bond motifs, see: Bernstein *et al.* (1995 ▶).
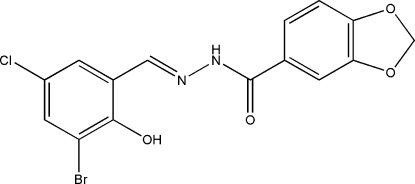


## Experimental

### 

#### Crystal data

C_15_H_10_BrClN_2_O_4_
*M* *_r_* = 397.61
Triclinic, 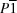

*a* = 9.769 (2) Å
*b* = 13.041 (3) Å
*c* = 13.251 (3) Å
α = 75.558 (2)°
β = 78.745 (2)°
γ = 76.527 (2)°
*V* = 1572.9 (6) Å^3^
*Z* = 4
Mo *K*α radiation
μ = 2.80 mm^−1^
*T* = 298 K
0.12 × 0.10 × 0.10 mm

#### Data collection

Bruker SMART CCD area-detector diffractometer
Absorption correction: multi-scan (*SADABS*; Sheldrick, 1996 ▶) *T*_min_ = 0.730, *T*_max_ = 0.767
8062 measured reflections
5662 independent reflections
3817 reflections with *I* > 2σ(*I*)
*R*_int_ = 0.019

#### Refinement

*R*[*F* ^2^ > 2σ(*F* ^2^)] = 0.048
*wR*(*F* ^2^) = 0.137
*S* = 1.01
5662 reflections
423 parameters
2 restraints
H atoms treated by a mixture of independent and constrained refinement
Δρ_max_ = 1.23 e Å^−3^
Δρ_min_ = −0.40 e Å^−3^

### 

Data collection: *SMART* (Bruker, 1998 ▶); cell refinement: *SAINT* (Bruker, 1998 ▶); data reduction: *SAINT*; program(s) used to solve structure: *SHELXS97* (Sheldrick, 2008 ▶); program(s) used to refine structure: *SHELXL97* (Sheldrick, 2008 ▶); molecular graphics: *SHELXTL* (Sheldrick, 2008 ▶); software used to prepare material for publication: *SHELXTL*.

## Supplementary Material

Crystal structure: contains datablock(s) global, I. DOI: 10.1107/S160053681201433X/sj5231sup1.cif

Structure factors: contains datablock(s) I. DOI: 10.1107/S160053681201433X/sj5231Isup2.hkl

Supplementary material file. DOI: 10.1107/S160053681201433X/sj5231Isup3.cml

Additional supplementary materials:  crystallographic information; 3D view; checkCIF report

## Figures and Tables

**Table 1:** Hydrogen-bond geometry (Å, °)

*D*—H⋯*A*	*D*—H	H⋯*A*	*D*⋯*A*	*D*—H⋯*A*
N2—H2⋯O4^i^	0.90 (1)	2.00 (2)	2.872 (4)	162 (5)
O3—H3⋯N3	0.82	1.92	2.637 (4)	145
O1—H1⋯N1	0.82	1.85	2.561 (4)	145

Symmetry code: (i) 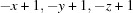.

**Fig. 1.**
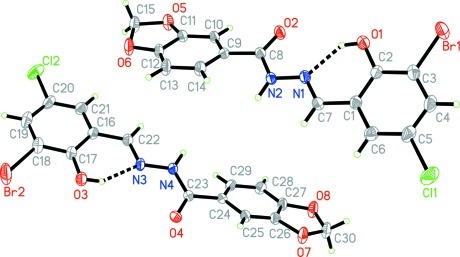
The molecular structure of the title compound, showing 30% probability displacement ellipsoids for the non-hydrogen atoms. Intramolecular hydrogen bonds are shown as dashed lines.

**Fig. 2.**
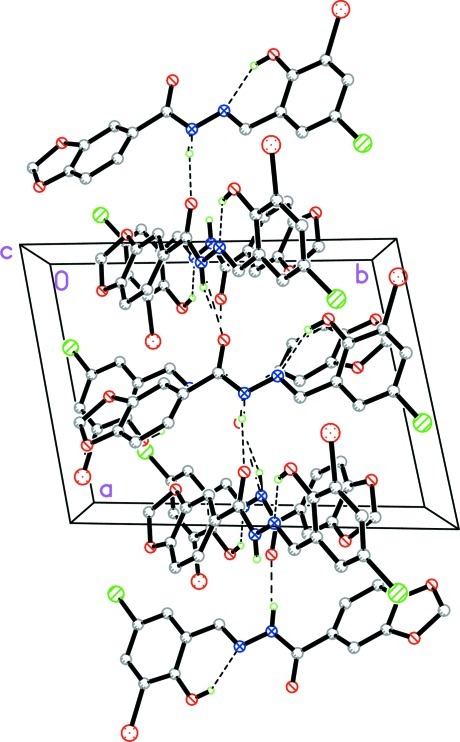
The packing diagram of the title compound, viewed along the c axis. Hydrogen bonds are shown as dashed lines.

## supplementary crystallographic information

### Comment

Hydrazone compounds derived from the condensation of aldehydes with hydrazides have been demonstrated to possess excellent biological activities (Zhong *et al.*, 2007; Raj *et al.*, 2007; Jimenez-Pulido *et al.*, 2008). Due to the easy synthesis of such compounds, a number of hydrazone compounds have been synthesized and structurally characterized (Yehye *et al.*, 2008; Fun *et al.*, 2008*a*,*b*; Yang *et al.*, 2008; Ejsmont *et al.*, 2008; Yang, 2006). Recently, we have reported several such compounds (Ban, 2010; Ban & Li, 2008*a*,*b*; Li & Ban, 2009*a*,*b*). We report here the crystal structure of the new title benzohydrazide derivative.

The asymmetric unit of the title hydrazone compound, Fig. 1, contains two independent molecules. The dihedral angles between the two benzene rings are 38.7 (3) and 24.3 (3)°, respectively. The molecules exist in *trans* configuration with respect to the central methylidene units. Intramolecular O1—H1···N1 and O3—H3···N3 contacts are observed forming S(6) rings (Bernstein *et al.*, 1995). In the crystal structure, molecules are linked through intermolecular N—H···O hydrogen bonds (Table 1), forming chains along the *a* axis, Fig. 2.

### Experimental

The title compound was prepared by refluxing 3-bromo-5-chlorosalicylaldehyde (1.0 mol, 0.23 g) with [3,4]dioxolebenzohydrazide (1.0 mol, 0.18 g) in methanol (50 ml). Excess methanol was removed from the mixture by distillation. A colourless solid product was filtered, and washed three times with methanol. Colourless block-shaped crystals of the title compound were obtained from a methanol solution of the compound by slow evaporation in air.

### Refinement

Atoms H2 and H4A were located in a difference Fourier map and refined isotropically, with the N—H distances restrained to 0.90 (1) Å. The remaining H atoms were placed in calculated positions (C—H = 0.93–0.97 Å, O—H = 0.82 Å) and refined as riding with *U*_iso_(H) = 1.2*U*_eq_(C) and 1.5 *U*_eq_(O). The structure contains solvent accessible voids of 78 Å^3^, which might accommodate a disordered methanol molecule. However, the effect of the presence of additional solvent was not investigated further.

### Crystal data

**Table:**

C_15_H_10_BrClN_2_O_4_	*Z* = 4
*M**_r_* = 397.61	*F*(000) = 792
Triclinic, *P*1	*D*_x_ = 1.679 Mg m^−^^3^
Hall symbol: -P 1	Mo *K*α radiation, λ = 0.71073 Å
*a* = 9.769 (2) Å	Cell parameters from 2572 reflections
*b* = 13.041 (3) Å	θ = 2.5–25.0°
*c* = 13.251 (3) Å	µ = 2.80 mm^−^^1^
α = 75.558 (2)°	*T* = 298 K
β = 78.745 (2)°	Block, colourless
γ = 76.527 (2)°	0.12 × 0.10 × 0.10 mm
*V* = 1572.9 (6) Å^3^	

### Data collection

**Table:**

Bruker SMART CCD area-detector diffractometer	5662 independent reflections
Radiation source: fine-focus sealed tube	3817 reflections with *I* > 2σ(*I*)
Graphite monochromator	*R*_int_ = 0.019
ω scans	θ_max_ = 25.5°, θ_min_ = 3.6°
Absorption correction: multi-scan (*SADABS*; Sheldrick, 1996)	*h* = −11→11
*T*_min_ = 0.730, *T*_max_ = 0.767	*k* = −15→14
8062 measured reflections	*l* = −16→15

### Refinement

**Table:**

Refinement on *F*^2^	Primary atom site location: structure-invariant direct methods
Least-squares matrix: full	Secondary atom site location: difference Fourier map
*R*[*F*^2^ > 2σ(*F*^2^)] = 0.048	Hydrogen site location: inferred from neighbouring sites
*wR*(*F*^2^) = 0.137	H atoms treated by a mixture of independent and constrained refinement
*S* = 1.01	*w* = 1/[σ^2^(*F*_o_^2^) + (0.0804*P*)^2^] where *P* = (*F*_o_^2^ + 2*F*_c_^2^)/3
5662 reflections	(Δ/σ)_max_ = 0.001
423 parameters	Δρ_max_ = 1.23 e Å^−^^3^
2 restraints	Δρ_min_ = −0.40 e Å^−^^3^

### Special details

**Table:**

Geometry. All e.s.d.'s (except the e.s.d. in the dihedral angle between two l.s. planes) are estimated using the full covariance matrix. The cell e.s.d.'s are taken into account individually in the estimation of e.s.d.'s in distances, angles and torsion angles; correlations between e.s.d.'s in cell parameters are only used when they are defined by crystal symmetry. An approximate (isotropic) treatment of cell e.s.d.'s is used for estimating e.s.d.'s involving l.s. planes.
Refinement. Refinement of *F*^2^ against ALL reflections. The weighted *R*-factor *wR* and goodness of fit *S* are based on *F*^2^, conventional *R*-factors *R* are based on *F*, with *F* set to zero for negative *F*^2^. The threshold expression of *F*^2^ > σ(*F*^2^) is used only for calculating *R*-factors(gt) *etc*. and is not relevant to the choice of reflections for refinement. *R*-factors based on *F*^2^ are statistically about twice as large as those based on *F*, and *R*- factors based on ALL data will be even larger.

### Fractional atomic coordinates and isotropic or equivalent isotropic displacement parameters (Å^2^)

**Table:**

	*x*	*y*	*z*	*U*_iso_*/*U*_eq_	
Br1	−0.32727 (5)	0.72347 (4)	1.11304 (4)	0.0705 (2)	
Br2	0.88064 (7)	−0.00317 (5)	0.23324 (6)	0.0941 (3)	
Cl1	0.21177 (17)	0.81156 (13)	1.05109 (10)	0.0840 (4)	
Cl2	0.35756 (17)	0.02462 (10)	0.11594 (10)	0.0753 (4)	
N1	0.0059 (3)	0.5383 (2)	0.8013 (2)	0.0388 (7)	
N2	0.0549 (3)	0.4803 (2)	0.7245 (2)	0.0388 (7)	
N3	0.5226 (3)	0.3717 (2)	0.3015 (2)	0.0395 (7)	
N4	0.4588 (3)	0.4720 (2)	0.3202 (3)	0.0404 (7)	
O1	−0.1949 (3)	0.6081 (2)	0.9379 (2)	0.0517 (7)	
H1	−0.1559	0.5706	0.8951	0.078*	
O2	−0.1698 (3)	0.4846 (2)	0.7053 (2)	0.0543 (7)	
O3	0.7314 (3)	0.2028 (2)	0.2949 (2)	0.0504 (7)	
H3	0.6932	0.2628	0.3057	0.076*	
O4	0.6578 (3)	0.4993 (2)	0.3652 (2)	0.0463 (6)	
O5	−0.0839 (3)	0.2765 (2)	0.4030 (2)	0.0548 (7)	
O6	0.1385 (3)	0.1716 (2)	0.4079 (3)	0.0657 (9)	
O7	0.4445 (3)	0.8606 (2)	0.4909 (2)	0.0552 (8)	
O8	0.2603 (3)	0.9451 (2)	0.3952 (3)	0.0622 (8)	
C1	0.0423 (4)	0.6396 (3)	0.9147 (3)	0.0409 (9)	
C2	−0.0981 (4)	0.6516 (3)	0.9635 (3)	0.0407 (9)	
C3	−0.1388 (5)	0.7108 (3)	1.0418 (3)	0.0486 (10)	
C4	−0.0453 (5)	0.7608 (3)	1.0691 (3)	0.0576 (11)	
H4	−0.0752	0.8024	1.1199	0.069*	
C5	0.0917 (6)	0.7474 (4)	1.0197 (3)	0.0557 (11)	
C6	0.1375 (5)	0.6876 (3)	0.9435 (3)	0.0492 (10)	
H6	0.2318	0.6793	0.9113	0.059*	
C7	0.0940 (4)	0.5775 (3)	0.8325 (3)	0.0389 (8)	
H7	0.1896	0.5668	0.8035	0.047*	
C8	−0.0431 (4)	0.4514 (3)	0.6826 (3)	0.0374 (8)	
C9	0.0141 (4)	0.3772 (3)	0.6091 (3)	0.0375 (8)	
C10	−0.0726 (4)	0.3719 (3)	0.5385 (3)	0.0378 (8)	
H10	−0.1616	0.4166	0.5342	0.045*	
C11	−0.0211 (4)	0.2988 (3)	0.4768 (3)	0.0383 (8)	
C12	0.1124 (4)	0.2353 (3)	0.4811 (3)	0.0481 (10)	
C13	0.1984 (5)	0.2380 (4)	0.5486 (4)	0.0615 (13)	
H13	0.2874	0.1931	0.5513	0.074*	
C14	0.1469 (4)	0.3115 (3)	0.6138 (3)	0.0511 (10)	
H14	0.2027	0.3164	0.6612	0.061*	
C15	0.0072 (5)	0.1843 (4)	0.3712 (4)	0.0587 (12)	
H15A	−0.0344	0.1207	0.4013	0.070*	
H15B	0.0218	0.1947	0.2950	0.070*	
C16	0.5024 (4)	0.2181 (3)	0.2431 (3)	0.0416 (9)	
C17	0.6426 (4)	0.1645 (3)	0.2551 (3)	0.0420 (9)	
C18	0.6890 (5)	0.0671 (3)	0.2240 (3)	0.0537 (11)	
C19	0.6042 (6)	0.0232 (3)	0.1819 (4)	0.0625 (12)	
H19	0.6384	−0.0424	0.1613	0.075*	
C20	0.4687 (5)	0.0778 (3)	0.1709 (3)	0.0518 (10)	
C21	0.4175 (5)	0.1738 (3)	0.2008 (3)	0.0486 (10)	
H21	0.3248	0.2097	0.1928	0.058*	
C22	0.4453 (4)	0.3235 (3)	0.2702 (3)	0.0432 (9)	
H22	0.3508	0.3557	0.2645	0.052*	
C23	0.5344 (4)	0.5333 (3)	0.3475 (3)	0.0359 (8)	
C24	0.4563 (4)	0.6435 (3)	0.3554 (3)	0.0339 (8)	
C25	0.4952 (4)	0.6931 (3)	0.4253 (3)	0.0376 (8)	
H25	0.5668	0.6581	0.4661	0.045*	
C26	0.4240 (4)	0.7935 (3)	0.4306 (3)	0.0395 (9)	
C27	0.3156 (4)	0.8472 (3)	0.3720 (3)	0.0416 (9)	
C28	0.2767 (4)	0.8009 (3)	0.3039 (3)	0.0453 (9)	
H28	0.2038	0.8368	0.2646	0.054*	
C29	0.3496 (4)	0.6983 (3)	0.2953 (3)	0.0411 (9)	
H29	0.3265	0.6652	0.2480	0.049*	
C30	0.3443 (5)	0.9591 (3)	0.4641 (4)	0.0557 (11)	
H30A	0.2843	0.9769	0.5273	0.067*	
H30B	0.3942	1.0177	0.4303	0.067*	
H2	0.146 (2)	0.472 (4)	0.693 (4)	0.080*	
H4A	0.3647 (15)	0.492 (4)	0.319 (4)	0.080*	

### Atomic displacement parameters (Å^2^)

**Table:**

	*U*^11^	*U*^22^	*U*^33^	*U*^12^	*U*^13^	*U*^23^
Br1	0.0662 (3)	0.0716 (3)	0.0670 (3)	−0.0066 (2)	0.0176 (2)	−0.0304 (2)
Br2	0.0815 (4)	0.0707 (4)	0.1334 (5)	0.0298 (3)	−0.0303 (4)	−0.0577 (4)
Cl1	0.1060 (11)	0.1103 (11)	0.0655 (7)	−0.0541 (9)	−0.0172 (7)	−0.0390 (7)
Cl2	0.1106 (11)	0.0657 (8)	0.0708 (7)	−0.0447 (7)	−0.0206 (7)	−0.0219 (6)
N1	0.0374 (17)	0.0426 (17)	0.0378 (16)	−0.0042 (14)	−0.0025 (13)	−0.0166 (14)
N2	0.0320 (16)	0.0453 (18)	0.0431 (17)	−0.0064 (14)	0.0003 (13)	−0.0223 (14)
N3	0.0398 (17)	0.0308 (16)	0.0480 (17)	0.0007 (13)	−0.0049 (14)	−0.0172 (14)
N4	0.0302 (16)	0.0333 (16)	0.0592 (19)	−0.0001 (13)	−0.0061 (14)	−0.0186 (14)
O1	0.0400 (15)	0.0580 (18)	0.0614 (18)	−0.0081 (13)	−0.0004 (13)	−0.0270 (14)
O2	0.0298 (14)	0.0656 (18)	0.0775 (19)	−0.0028 (13)	−0.0023 (13)	−0.0440 (16)
O3	0.0434 (16)	0.0454 (16)	0.0669 (17)	0.0004 (12)	−0.0123 (13)	−0.0256 (14)
O4	0.0269 (14)	0.0460 (15)	0.0688 (17)	−0.0012 (11)	−0.0088 (12)	−0.0211 (13)
O5	0.0484 (16)	0.0596 (18)	0.0627 (17)	0.0092 (14)	−0.0202 (14)	−0.0336 (15)
O6	0.0516 (18)	0.0624 (19)	0.098 (2)	0.0120 (15)	−0.0205 (16)	−0.0581 (18)
O7	0.0619 (18)	0.0482 (16)	0.0630 (17)	0.0101 (14)	−0.0245 (15)	−0.0329 (14)
O8	0.066 (2)	0.0450 (16)	0.082 (2)	0.0141 (14)	−0.0274 (16)	−0.0353 (15)
C1	0.046 (2)	0.042 (2)	0.0371 (19)	−0.0084 (17)	−0.0055 (16)	−0.0121 (16)
C2	0.048 (2)	0.036 (2)	0.041 (2)	−0.0087 (17)	−0.0067 (17)	−0.0116 (16)
C3	0.058 (3)	0.042 (2)	0.041 (2)	−0.0035 (19)	−0.0014 (18)	−0.0106 (18)
C4	0.085 (3)	0.052 (3)	0.040 (2)	−0.021 (2)	0.000 (2)	−0.0193 (19)
C5	0.081 (3)	0.060 (3)	0.037 (2)	−0.025 (2)	−0.015 (2)	−0.0151 (19)
C6	0.056 (3)	0.055 (2)	0.042 (2)	−0.017 (2)	−0.0079 (18)	−0.0140 (19)
C7	0.0341 (19)	0.045 (2)	0.0378 (19)	−0.0039 (17)	−0.0038 (15)	−0.0134 (17)
C8	0.0309 (19)	0.040 (2)	0.043 (2)	−0.0047 (16)	−0.0042 (15)	−0.0152 (16)
C9	0.0297 (18)	0.0350 (19)	0.050 (2)	−0.0051 (15)	−0.0019 (16)	−0.0168 (16)
C10	0.0310 (18)	0.040 (2)	0.0422 (19)	−0.0004 (16)	−0.0045 (15)	−0.0155 (16)
C11	0.0341 (19)	0.041 (2)	0.0406 (19)	−0.0023 (16)	−0.0057 (15)	−0.0144 (16)
C12	0.040 (2)	0.042 (2)	0.068 (3)	0.0003 (18)	−0.0067 (19)	−0.031 (2)
C13	0.042 (2)	0.053 (3)	0.102 (4)	0.014 (2)	−0.026 (2)	−0.047 (3)
C14	0.039 (2)	0.051 (2)	0.072 (3)	0.0023 (18)	−0.022 (2)	−0.030 (2)
C15	0.058 (3)	0.053 (3)	0.072 (3)	0.007 (2)	−0.019 (2)	−0.037 (2)
C16	0.046 (2)	0.036 (2)	0.045 (2)	−0.0074 (17)	−0.0051 (17)	−0.0129 (16)
C17	0.049 (2)	0.036 (2)	0.042 (2)	−0.0043 (17)	−0.0066 (17)	−0.0146 (17)
C18	0.063 (3)	0.042 (2)	0.054 (2)	0.004 (2)	−0.011 (2)	−0.0182 (19)
C19	0.086 (4)	0.040 (2)	0.064 (3)	−0.005 (2)	−0.008 (2)	−0.023 (2)
C20	0.073 (3)	0.041 (2)	0.049 (2)	−0.021 (2)	−0.008 (2)	−0.0163 (19)
C21	0.049 (2)	0.045 (2)	0.056 (2)	−0.0101 (19)	−0.0122 (19)	−0.0141 (19)
C22	0.037 (2)	0.039 (2)	0.057 (2)	−0.0028 (17)	−0.0084 (17)	−0.0186 (18)
C23	0.033 (2)	0.0370 (19)	0.0387 (19)	−0.0071 (16)	0.0000 (15)	−0.0133 (16)
C24	0.0285 (18)	0.0319 (18)	0.0411 (19)	−0.0044 (14)	−0.0017 (15)	−0.0112 (15)
C25	0.0328 (19)	0.039 (2)	0.0391 (19)	0.0005 (16)	−0.0055 (15)	−0.0121 (16)
C26	0.039 (2)	0.043 (2)	0.0391 (19)	−0.0039 (17)	−0.0061 (16)	−0.0171 (17)
C27	0.041 (2)	0.033 (2)	0.050 (2)	0.0025 (16)	−0.0072 (17)	−0.0157 (17)
C28	0.046 (2)	0.040 (2)	0.053 (2)	−0.0036 (18)	−0.0173 (18)	−0.0127 (18)
C29	0.042 (2)	0.036 (2)	0.051 (2)	−0.0074 (16)	−0.0118 (18)	−0.0136 (17)
C30	0.064 (3)	0.046 (2)	0.062 (3)	0.008 (2)	−0.019 (2)	−0.031 (2)

### Geometric parameters (Å, º)

**Table:**

Br1—C3	1.888 (4)	C7—H7	0.9300
Br2—C18	1.896 (4)	C8—C9	1.484 (5)
Cl1—C5	1.752 (5)	C9—C14	1.381 (5)
Cl2—C20	1.761 (4)	C9—C10	1.403 (5)
N1—C7	1.274 (5)	C10—C11	1.354 (5)
N1—N2	1.365 (4)	C10—H10	0.9300
N2—C8	1.359 (5)	C11—C12	1.376 (5)
N2—H2	0.899 (10)	C12—C13	1.352 (6)
N3—C22	1.268 (5)	C13—C14	1.392 (6)
N3—N4	1.372 (4)	C13—H13	0.9300
N4—C23	1.355 (5)	C14—H14	0.9300
N4—H4A	0.898 (10)	C15—H15A	0.9700
O1—C2	1.343 (5)	C15—H15B	0.9700
O1—H1	0.8200	C16—C21	1.379 (5)
O2—C8	1.216 (4)	C16—C17	1.405 (6)
O3—C17	1.338 (5)	C16—C22	1.464 (5)
O3—H3	0.8200	C17—C18	1.381 (5)
O4—C23	1.231 (4)	C18—C19	1.373 (6)
O5—C11	1.370 (4)	C19—C20	1.365 (7)
O5—C15	1.422 (5)	C19—H19	0.9300
O6—C12	1.379 (4)	C20—C21	1.362 (6)
O6—C15	1.419 (5)	C21—H21	0.9300
O7—C26	1.392 (4)	C22—H22	0.9300
O7—C30	1.434 (5)	C23—C24	1.481 (5)
O8—C27	1.351 (4)	C24—C29	1.388 (5)
O8—C30	1.410 (5)	C24—C25	1.405 (5)
C1—C2	1.388 (5)	C25—C26	1.343 (5)
C1—C6	1.390 (6)	C25—H25	0.9300
C1—C7	1.459 (5)	C26—C27	1.385 (5)
C2—C3	1.389 (5)	C27—C28	1.355 (5)
C3—C4	1.383 (6)	C28—C29	1.383 (5)
C4—C5	1.363 (7)	C28—H28	0.9300
C4—H4	0.9300	C29—H29	0.9300
C5—C6	1.372 (6)	C30—H30A	0.9700
C6—H6	0.9300	C30—H30B	0.9700
			
C7—N1—N2	118.6 (3)	C13—C14—H14	119.6
C8—N2—N1	117.5 (3)	O6—C15—O5	107.1 (3)
C8—N2—H2	120 (3)	O6—C15—H15A	110.3
N1—N2—H2	121 (3)	O5—C15—H15A	110.3
C22—N3—N4	115.6 (3)	O6—C15—H15B	110.3
C23—N4—N3	120.5 (3)	O5—C15—H15B	110.3
C23—N4—H4A	122 (3)	H15A—C15—H15B	108.6
N3—N4—H4A	117 (3)	C21—C16—C17	120.1 (3)
C2—O1—H1	109.5	C21—C16—C22	118.5 (3)
C17—O3—H3	109.5	C17—C16—C22	121.3 (3)
C11—O5—C15	105.9 (3)	O3—C17—C18	119.3 (4)
C12—O6—C15	106.2 (3)	O3—C17—C16	123.4 (3)
C26—O7—C30	105.0 (3)	C18—C17—C16	117.3 (4)
C27—O8—C30	106.7 (3)	C19—C18—C17	122.5 (4)
C2—C1—C6	120.0 (3)	C19—C18—Br2	119.5 (3)
C2—C1—C7	121.7 (3)	C17—C18—Br2	117.9 (3)
C6—C1—C7	118.3 (4)	C20—C19—C18	118.7 (4)
O1—C2—C1	122.5 (3)	C20—C19—H19	120.7
O1—C2—C3	119.2 (4)	C18—C19—H19	120.7
C1—C2—C3	118.3 (4)	C21—C20—C19	121.2 (4)
C4—C3—C2	121.9 (4)	C21—C20—Cl2	118.9 (4)
C4—C3—Br1	119.1 (3)	C19—C20—Cl2	119.9 (3)
C2—C3—Br1	119.0 (3)	C20—C21—C16	120.2 (4)
C5—C4—C3	118.3 (4)	C20—C21—H21	119.9
C5—C4—H4	120.8	C16—C21—H21	119.9
C3—C4—H4	120.8	N3—C22—C16	121.1 (3)
C4—C5—C6	121.8 (4)	N3—C22—H22	119.5
C4—C5—Cl1	119.3 (3)	C16—C22—H22	119.5
C6—C5—Cl1	118.8 (4)	O4—C23—N4	122.4 (3)
C5—C6—C1	119.6 (4)	O4—C23—C24	122.8 (3)
C5—C6—H6	120.2	N4—C23—C24	114.7 (3)
C1—C6—H6	120.2	C29—C24—C25	120.1 (3)
N1—C7—C1	118.8 (3)	C29—C24—C23	122.1 (3)
N1—C7—H7	120.6	C25—C24—C23	117.8 (3)
C1—C7—H7	120.6	C26—C25—C24	116.8 (3)
O2—C8—N2	121.2 (3)	C26—C25—H25	121.6
O2—C8—C9	122.8 (3)	C24—C25—H25	121.6
N2—C8—C9	115.9 (3)	C25—C26—C27	123.0 (3)
C14—C9—C10	121.0 (3)	C25—C26—O7	128.1 (3)
C14—C9—C8	120.6 (3)	C27—C26—O7	108.9 (3)
C10—C9—C8	118.3 (3)	O8—C27—C28	128.5 (3)
C11—C10—C9	117.0 (3)	O8—C27—C26	110.4 (3)
C11—C10—H10	121.5	C28—C27—C26	121.1 (3)
C9—C10—H10	121.5	C27—C28—C29	117.4 (3)
C10—C11—O5	128.8 (3)	C27—C28—H28	121.3
C10—C11—C12	121.2 (3)	C29—C28—H28	121.3
O5—C11—C12	109.9 (3)	C28—C29—C24	121.6 (3)
C13—C12—C11	123.2 (3)	C28—C29—H29	119.2
C13—C12—O6	128.1 (3)	C24—C29—H29	119.2
C11—C12—O6	108.7 (3)	O8—C30—O7	108.6 (3)
C12—C13—C14	116.6 (4)	O8—C30—H30A	110.0
C12—C13—H13	121.7	O7—C30—H30A	110.0
C14—C13—H13	121.7	O8—C30—H30B	110.0
C9—C14—C13	120.9 (4)	O7—C30—H30B	110.0
C9—C14—H14	119.6	H30A—C30—H30B	108.4

### Hydrogen-bond geometry (Å, º)

**Table:**

*D*—H···*A*	*D*—H	H···*A*	*D*···*A*	*D*—H···*A*
N2—H2···O4^i^	0.90 (1)	2.00 (2)	2.872 (4)	162 (5)
O3—H3···N3	0.82	1.92	2.637 (4)	145
O1—H1···N1	0.82	1.85	2.561 (4)	145

Symmetry code: (i) −*x*+1, −*y*+1, −*z*+1.
